# Venous access devices (Review)

**DOI:** 10.3892/mi.2025.241

**Published:** 2025-05-13

**Authors:** Mohammad Abdulelah, Omar Haider, Hussein Abdulelah, Kevin T. Jamouss, Thomas L. Higgins

**Affiliations:** 1 Department of Medicine, University of Massachusetts Chan Medical School - Baystate, Springfield, MA 01199, USA; 2 Department of Medicine, University of Jordan, Amman 11942, Jordan; 3 Division of Pulmonary and Critical Care Medicine, University of Massachusetts Chan Medical School - Baystate, Springfield, MA 01199, USA

**Keywords:** venous access devices, peripheral intravenous catheters, non-tunneled central venous access catheters, tunneled central venous access catheters

## Abstract

Venous access devices can be categorized based on the termination site of the tip of the catheter into central and peripheral access devices. Selecting the type of venous access device depends on various factors, including the condition of the patient, the anticipated duration of therapy, the use of vesicant or hyperosmolar therapies and the potential risk of complications. Peripheral intravenous catheters provide adequate venous access in the majority of hospitalized patients; however, their use is associated with high failure rates. Non-tunneled central venous access catheters are typically used in critically ill patients and are ideally suited for short-term use, while tunneled central catheters are utilized in those who require long-term intravenous therapy due to their extended dwell times. Each of these devices has unique characteristics requiring an in-depth understanding of the indications and current evidence-based recommendations to optimize their use. The present narrative review aimed to describe the different types of venous access devices and recommendations for their use based on current evidence-based recommendations. A narrative review of the literature was performed based on searches of the PubMed and Google Scholar database from 1990 through November 1, 2024. The type of device used, the insertion site, patient characteristics and clinical needs, and the risk of complications are factors that a provider needs to consider when ordering the placement of a venous access device are discussed. The present review also discusses the prevention of negative adverse events, such as bloodstream infections and thrombosis, associated with specific devices. In addition, current evidence-based recommendations for device selection and indications for use are described. The present narrative review provides a detailed examination of venous access devices that are essential in the care and treatment of patients. Since venous access is associated with inherent risks, device selection and meticulous post-insertion care are paramount in ensuring patient safety and successful therapy.

## 1. Introduction

The selection of the appropriate venous access device often depends on the condition of the patient, the duration of therapy, the nature of the infusate, the potential risk of complications and whether venous access is elective or emergent (1,2). Each access device poses certain benefits over others, and healthcare providers often select them based on clinical factors, availability and anticipated complications (Table I) (1). For instance, peripheral intravenous catheters (PIVCs) are suitable for short-term therapies; however, they are less durable than more invasive centrally inserted catheters. Peripherally inserted central catheters (PICCs) have prolonged dwelling times, rendering them more suitable for long-term therapies, such as long-term antibiotic treatment (3). Centrally inserted central catheters (CICCs) are often preferred for patients in critical care settings or during surgical procedures, given the ease of insertion and larger lumens.

The present narrative review discusses the different types of venous access devices, including PIVCs, long peripheral catheters, midline catheters, CICCs and implantable ports. The aim of the present review was to provide a comprehensive understanding of the indications and current evidence-based recommendations for venous access devices to assist junior healthcare providers in making informed decisions through delineating the appropriate clinical conditions in which each access device is indicated. Vascular access in specific patient population, such as those receiving chemotherapy, hemodialysis or undergoing hemodynamic monitoring are also discussed. In addition, some of the common adverse events and complications, such as infections and thrombosis associated with venous catheters are discussed.

## 2. Data collection methods

For the purpose of the present narrative review article, the ‘best-evidence synthesis’ set forth by Slavin (4) was followed and the PubMed and Google Scholar databases were searched to identify articles from 1990 through November 1, 2024. The following medical subject headings were used: Venous access devices, venous catheters, peripheral intravenous catheters, long peripheral catheters, midline catheters, central venous access, peripherally inserted central catheters, ultrasound guided venous access, central line associated blood stream infection, catheter related blood stream infection and catheter associated thrombosis. The snowball technique was implemented, whereas the bibliographies of the retrieved articles were analyzed for other relevant articles. Search results were filtered for articles in the English language only. Of note, two authors (MA and KTJ) performed the search. Of the 882 articles identified, 94 articles were selected. Article selection involved screening the titles and abstracts for eligibility, followed by a full-text review to assess relevance to venous access devices. Disagreements regarding study inclusion were resolved by a consensus between the authors in consultation with a third author (HA). For inclusion, only peer reviewed articles were selected. Articles were selected based on relevance to current clinical practice educational value and the degree of evidence. Randomized clinical trials, large longitudinal observational studies, and more recent articles were prioritized. The artwork was independently created by the authors.

## 3. Peripheral venous access

### Peripheral intravenous catheters

Short peripheral intravenous catheters (PIVCs), commonly referred to as ‘peripheral IVs’ are a mainstay in healthcare. Common insertion sites include the median antecubital, cephalic or basilic veins (2). The adoption of ultrasound-guided peripheral intravenous access has markedly reduced the number of insertion attempts and has improved the success rates by 2- or 3-fold when compared to the traditional blind technique (2,5,6). A universal Birmingham gauge-specific coloring system has been adopted, which allows for the rapid identification of PIVC size, which ranges from 14 to 30 gauge (5). Accordingly, flow rates differ considerably, from 13 ml/min in a 26-gauge catheter to a robust 270 ml/min in a 14-gauge catheter, rendering a wide gauge PIVC an ideal option for early resuscitation efforts. However, one must also acknowledge that such catheters have their limitations in resuscitation; for instance, catheters <23 gauge are associated with hemolysis when blood is transfused (7). The benefits of short PIVCs include the ease of insertion, the immediate initiation of therapy and cost-effectiveness. However, the dwell time of short PIVCs is relatively limited, typically between 72 to 96 h, after which the risk of complications, such as phlebitis and mechanical complications significantly increases (2,8). Notably, ~19% of patients develop phlebitis (9). It is recommended that the short PIVC be removed at the first sign of phlebitis (5).

The microbial colonization of peripheral catheters can occur within 24 h of placement; however, this does not necessarily reflect a clinical infection (8). Although the incidence of short PIVC-associated infections is underreported, the incidence is reported to be at ~2% (10). One factor that affects the risk of short PIVC-associated infections is the type of material catheters are made from. Catheters made from teflon or polyurethane have lower rates of infection than those made of polyvinyl chloride or polyethylene (11).

Short PIVCs are also susceptible to mechanical complications, such as dislodgement, infiltration, extravasation, or occlusion (2,5,8-10). Infiltration refers to the unintentional leakage of a non-vesicant solution into the surrounding tissue, while extravasation is the leakage of a vesicant solution. Extravasation and infiltration are caused by several factors, including the migration of the catheter out of the vein over time, incomplete threading into the vein during insertion, or passing through the vein and out into the surrounding tissues (5,12). The management of infiltration and extravasation usually depends on the pharmacological properties of the infused solution and specific institutional guidelines (12,13)

Integrated short peripheral catheters have been introduced to overcome the mechanical complications associated with short PIVC. Integrated catheters are described as closed system catheters that are winged, non-ported, and equipped with preassembled extension and preassembled needle-free connector that have added safety mechanisms against needle stick injury prevention (14). These catheters have a prolonged dwell time of 6 days when compared to standard PIVC, as well as a statistically significant reduction in the risk of catheter failure as and occlusion (15).

### Long peripheral catheters

Long peripheral catheters (LPCs) are 6-15 cm in length and are typically inserted into the forearm or upper arm and the distal catheter tip terminates before reaching the axilla (16). They are recommended in patients with difficult IV access due to the ease of their insertion, where it has been reported that average insertion time ranges from 8 to 16.8 min (17). Dwell time is recommended to not exceed 4 weeks (18). Such catheters are a novelty in the current era, and significant confusion exists regarding their nomenclature (16). Nonetheless, LPCs had lower failure rates and longer dwell times when compared to PIVCs (17).

### Midline catheters

**Figure 1:**
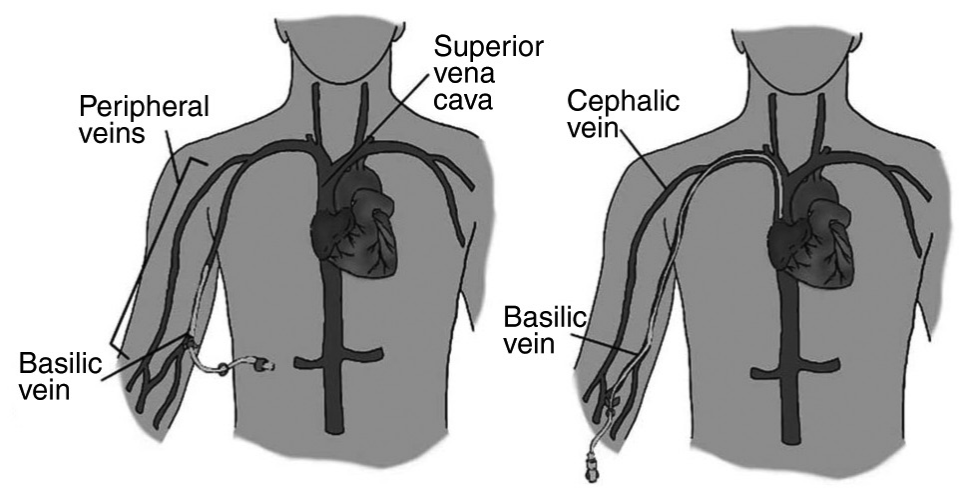
The image on the left panel portrays a midline inserted in the peripheral basilic vein with the tip located within the peripheral venous system. In general, midlines can be placed in basilic, brachial or cephalic veins. The image on the right panel portrays a PICC line with the access site being in the basilic vein. PICCs are advanced until the tip terminates in the cavoatrial junction. Catheter length also varies, whereas PICCs are about double the length. PICC, peripherally inserted central catheter.

Midline catheters (MCs) are peripheral venous access devices typically ranging from 15 to 25 cm in length (16). They are inserted into upper arm with the catheter tip positioned at or slightly below the level of the axillary vein, as illustrated in Fig. 1 (2,19). Such catheters are used primarily in patients requiring intermediate term intravenous therapy of around 6-14 days (20). Their prevalence is dependent on institutional protocols, clinician preferences and healthcare settings (21). Such catheters access larger veins, and are therefore advantageous for those with difficult venous access.

Compared to PIVCs, MCs are associated with a lower incidence of phlebitis, thereby enhancing patient satisfaction and decreasing the need for catheter replacement (21). The rate of catheter-related bloodstream infections (CRBSIs) in MCs aligns with that seen in PIVCs but is significantly lower than that of CICCs (21). Additionally, patients with difficult access to healthcare or poor adherence to infection control protocols might not be ideal candidates for an MC (22).

Despite their advantages, MCs have specific limitations. They are not recommended for the administration of vesicant or highly irritating medications, such as chemotherapy agents, which could potentially damage the peripheral veins (2,21). A notable decline in their use has been noted after the introduction of PICCs, which offer longer dwell times and lower overall complication rates (23). However, midlines were associated with a lower risk of bloodstream infection and occlusion compared with PICC when duration of use is less than 10 days (21). Yet, one must acknowledge that the lower complication risk of the midline is likely due to their lower dwell time when compared to PICCs rather than an inherit catheter property.

## 4. Central venous access

Central venous access devices (CVADs) are indwelling catheters that are inserted into large, central veins. Catheter diameters are measured using the French gauge system, which has uniform increments of 1/3 mm between sizes, unlike the non-uniform Birmingham gauge system developed for industrial wire sizes, and adapted for medical use to describe the outer diameter of the catheter. A lower French-gauge number indicates a larger catheter diameter.

As per the World Conference on Vascular Access (WoCoVA) definitions, the position of the tip of the catheter is inside the superior vena cava, inside the right atrium, or inside the inferior vena cava (1) The types of CVADs include tunneled and non-tunneled CICCs, PICCs and implantable ports. Notably, the terms CICC and Centrally Inserted Venous Catheter (CIVC) are used interchangeably depending on the area of practice. This type of access is typically utilized when patients require multiple simultaneous intravenous therapies and when central venous system access is required, such as in dialysis. Other indications include the need for recurrent blood sample collection when peripheral sampling is challenging, the administration of parenteral nutrition, plasmapheresis and central venous pressure monitoring (24).

Such devices are mainly placed at bedside under sterile conditions. Following catheter implantation, a chest radiograph is typically performed to verify the correct positioning of the catheter and to identify any potential complications, such as pneumothorax or mispositioning (24). However, the experience of one center suggests against using postprocedural X-rays, given the exceedingly rare complications and excessively high costs (25). The intraprocedural assessment of the position of the catheter tip can be performed through fluoroscopy, intracavitary ECG or echocardiography (26-28).

One commonly encountered indication for central access has been vasopressor medication administration. Policies requiring CVAD vary significantly across health care systems, and mainly dependent on agents to be infused, availability of skillset, and anticipated duration of therapy. A previous state-wide study performed across hospitals in Michigan revealed that 73% of hospitals required or preferred central access prior to initiation of vasopressor medications (29). Recently, the safe peripheral administration of vasopressors has been demonstrated and was associated with expedited vasopressor initiation (30). Another multicenter prospective study revealed that implementing a protocol for peripheral vasopressor administration leads to avoidance of requiring central access in 51.6% (31). Peripheral vasopressor administration up to 24 h has been shown to be safe with a low incidence of extravasation (32).

### PICC

A PICC is a slender (2-6 French) catheter ~50 to 60 cm in length that can have up to three lumens (33). It is usually inserted percutaneously with ultrasound or fluoroscopy guidance into the basilic or cephalic vein in the upper arm, as shown in Fig. 1 (19,33).

Due to the length of the catheter, PICCs can also be inserted in more distal veins. For instance, the catheterization of the great saphenous vein has been reported (34). PICCs reduce the procedural burden on physicians, as specially trained nurses can commonly perform the insertion at the patient's bedside, often utilizing ultrasound or fluoroscopy guidance (33). Their popularity has grown due to the potential to reduce hospitalization costs by shortening the length of hospitalization stay of patients by allowing for home intravenous therapy (2,33). These devices are indicated in patients requiring several weeks to 6 months of intravenous therapy; notably, experts recommend against their use when the proposed duration of use is <5 days (20). A contraindication is active IV substance use disorder given the concerns of inappropriate use upon discharge. However, mortality and catheter-related adverse events in this group have been found to be comparable to non-users, although, high quality evidence to inform practice is limited (35).

A commonly encountered practice is requesting multi-lumen PICCs in anticipation of future IV access requirements, although these are associated with a higher risk of complications compared to single-lumen PICCs (36).

### CICCs

CICCs can be categorized based on whether they are tunneled or non-tunneled (2,37). They are directly inserted into central veins without traveling through the subcutaneous tissue, hence the name ‘non-tunneled’. Conversely, tunneled catheters travel under the skin and terminate away from the venous access site, which decreases the risk of infection and ensures a more secure access point (2,37). The introduction of subcutaneously anchored securement devices has decreased the risk of catheter dislodgement to <3%; however, these devices have not been shown to reduce the risk of venous thrombosis or infections (38). These devices have also been used to anchor PICC and midline catheters (39). The duration of use for CICCs can vary depending on the catheter type, its intended purpose and patient-specific factors (2,24,33,37).

*Non-tunneled central venous catheters.* Non-tunneled central catheters are commonly referred to as central lines and are typically utilized in emergency departments, operating rooms, and critical care settings (2,24). These catheters are relatively straightforward to insert at the bedside, with ultrasound guidance being standard of care (6). Intended dwell time ranges from 3 days for femoral access to 2 weeks for jugular access; however, prompt removal is key as ~3% of patients develop one or more serious complications when catheters are left for ≥3 days (40,41). They are categorized upon the site of venous access as follows:

*a) Internal jugular vein*. Access to the internal jugular vein (IJV) provides several advantages. These include a reduced risk of pneumothorax compared to subclavian access, a short distance from skin to vessel and enhanced visualization of the needle path (42,43). The Trendelenburg position has been described as the standard of care during insertion due to the increase in IJV size, which ultimately decreases risk of developing complications; however, that compromises comfort in conscious patients (44). A previous study and a recent metanalysis revealed that further inclination from 10˚ does not statistically benefit IJV size (45,46). The IJV is the preferred access site for cardiac surgery and any procedure where the chest will be in the sterile field. However, using the IJV for venous access increases the risk of developing immediate complications, such as arterial puncture due the fact that the trajectory of the IJV often overlies the carotid artery. Potential nerve injury can also arise due to the proximity of the sympathetic chain. Although rare, Horner's syndrome has been reported during the placement of CICCs in the IJV (47,48). In addition, the risk of iatrogenic pneumothorax with IJV placement is extremely low in the era of ultrasound, at around 0.1% (49). When compared to the subclavian vein, the IJV imposes a higher risk of infection and thrombus formation (50).

*b) Femoral vein*. Femoral vein access with traditional catheters is classified as peripheral access as the length of most catheters is ~20 cm, which leaves the tip of the catheter located an iliac vein (either external or common) (1). Therefore, Annetta *et al* (51) suggested utilizing the term ‘femorally inserted central catheter’ whenever catheters culminate in the central circulation. Femoral vein access is often selected in emergency settings due to its easy accessibility and large caliber, facilitating swift catheter placement (24). However, it carries a heightened risk of infection and deep vein thrombosis compared to other sites (52). One way to mitigate infectious risks includes mid-thigh exit sites with pseudo-tunneling (53). Ideally, femoral catheters placed in emergency settings should be removed and replaced within 48 h of insertion (51).

**Figure 2:**
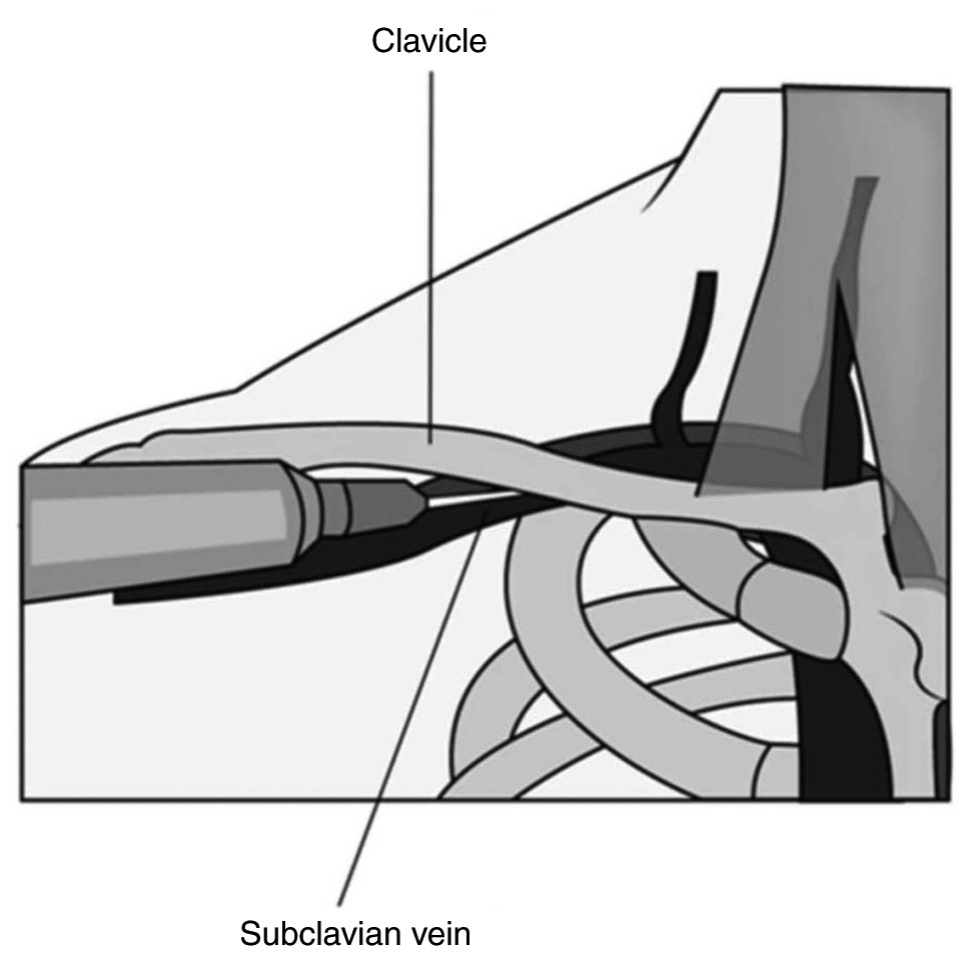
Subclavian vein venipuncture indicating the infraclavicular position of the needle during blind technique subclavian vein cannulation.

*c) Subclavian and thoracic axillary veins*. Subclavian vein access is often favored in patients requiring long-term catheterization. It has the lowest infection and thrombosis rates among central catheter sites but potentially has the highest rates of venous stenosis (24). Right-sided subclavian central catheters are associated with a lower incidence of pneumothorax, but an increased risk of catheter malposition, hemothorax and subclavian artery puncture due to the anatomical position of the subclavian vein (Fig. 2) (54). The catheterization of the supraclavicular tributary of the subclavian vein, when compared to the IJV, increased the first-attempt success proportion [relative risk (RR), 1.22; 95% confidence interval (CI), 1.06-1.40] (55). Historically, ultrasound guidance was linked with worse outcomes when compared to landmark or blind technique, however, recently published studies revealed lower complication rates and higher success rate with ultrasound guided placement when compared to blind technique (56). Nonetheless, a suitable alternative has been the long axillary vein as it resides outside the thoracic cavity and is not hindered by the clavicle promoting easier ultrasound visualization. A recent metanalysis of five randomized controlled trials compared blind technique subclavian catheterization to ultrasound guided axillary vein catheterization (57). There was a statistically significant increase the in overall catheterization success rates (RR, 1.09; 95% CI, 1.04-1.15; P<0.01) and a decrease in adverse events, including the risk of arterial puncture (RR, 0.18; 95% CI, 0.06-0.55; P<0.01) pneumo-and hemothorax (RR, 0.12; 95% CI, 0.02-0.64; P=0.01). However, all five studies in the metanalysis compared blind technique subclavian catheterization to US guided axillary vein (57).

*Tunneled central venous catheters.* These catheters are indicated for patients who require long term IV access. The exit site is in one location and is tunneled under the skin away from the point of venous entry (2,37). Tunneled catheters are typically inserted under ultrasound or fluoroscopy guidance and are positioned on the chest wall to facilitate catheter care. A unique feature of tunneled catheters is the anchoring Dacron cuff that facilitates internal fixation once tissue ingrowth occurs. Lower CRBSI rates for tunneled central catheters, when compared to PICCs, have been reported, and they have a longer time to the first infection (58). These catheters can be left in place for an extended period, ranging from several weeks to months. Potential complications of tunneled catheters include thrombosis, pneumothorax, extravasation, external fracture, and CLABSIs. Rare complications that may occur include air embolism and pinch-off syndrome (59). Commonly encountered tunneled catheters include Hickman, Broviac, Hohn and Groshong catheters.

### Special patient populations. Oncology patients and implantable ports

**Figure 3:**
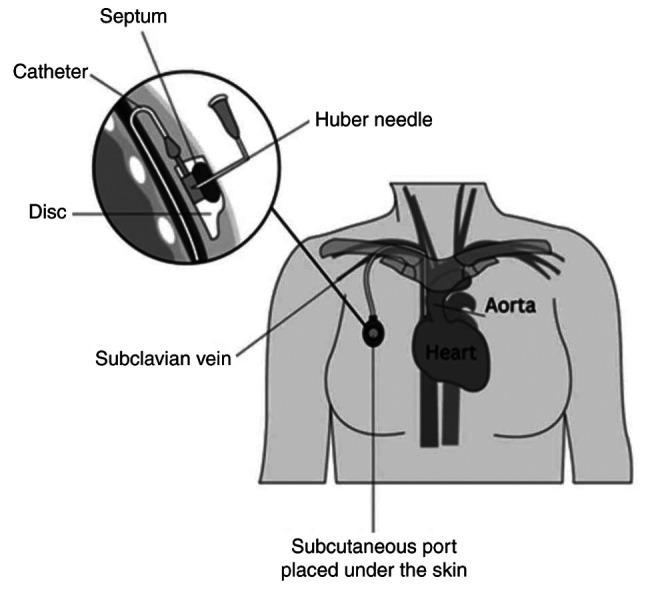
Subcutaneously implanted port on the anterior chest wall. The catheter is tunneled under the skin and accesses the subclavian vein.

Implantable port catheters, known simply as ‘ports’, provide superior patient comfort and mobility compared to external catheters (2). Despite their extra associated costs, they are extensively used in the field of oncology for administration of chemotherapy as they have been proven to improve patient satisfaction and quality of life (60). Given their placement beneath the skin, patients can engage in daily activities, such as showering and swimming without fear of disrupting the device. Placement of a port involves a surgical procedure, performed under either local or general anesthesia (61). During this procedure, a subcutaneous pocket is created in the chest wall and the catheter is typically tunneled into the jugular vein. Other potential sites for cannulation include the subclavian, cephalic, and innominate veins (Fig. 3) (62).

A more cosmetically appealing approach has been arm ports, which is appealing to some patients such as those undergoing therapy for breast cancer especially if they are requiring radiotherapy, flap transfer, or reconstructive surgeries (63). Furthermore, femoral placement of ports has been described in patients with bilateral breast cancer or hindered neck and chest accessibility (64,65). Notably, implanted ports provide a reduced risk of infectious and mechanical complications when compared to either PICC or tunneled catheters (60,66).

*Hemodialysis patients and dialysis catheters.* Central venous catheters offer a crucial alternative in hemodialysis patients without a functioning arteriovenous (AV) fistula or graft (67). This form of access becomes particularly relevant when urgent hemodialysis needs to be performed. Patients initiating hemodialysis with non-tunneled catheters face significantly higher infection rates compared to those using tunneled hemodialysis catheters (67,68). It is important to note that while CICCs can provide immediate access to hemodialysis, they should not be viewed as a long-term solution as the recommended duration of use is <2 weeks (69) facilitate life-saving treatment; however, their use should be minimized to decrease the risk of central venous stenosis or occlusion, a severe complication that can compromise future permanent access options (68). In terms of site selection for venous access, the National Kidney Foundation's Kidney Disease Outcomes Quality Initiative (KDOQI) guidelines suggest a hierarchy of choice. The IJV is the most recommended site, followed by external jugular, femoral veins and the subclavian vein is the least preferred due to the inherent high risk of stenosis. Right sided insertion is preferable due to more direct anatomy in the absence of contraindications, such as central stenosis or previous pacemaker insertion (69).

In those who require long term dialysis and do not have a mature arteriovenous fistula, tunneled catheters are the preferred venous access. In the most recent statement of the KDOQI, no maximal dwell time was recommended; rather, the indefinite use of tunneled catheters can be pursued if they are deemed to be the most appropriate permanent dialysis access (69). Two separate catheters can be inserted side by side (Tesio catheters) or a single dual-lumen catheter (Permcath) can be used (70).

*Hemodynamically monitored patients and Swan-Ganz catheters.* The Swan-Ganz catheter is a pulmonary artery catheter used to monitor the hemodynamic status of critical patients (71). It is used to evaluate right-sided cardiac filling pressures, estimation of cardiac output, intracardiac shunt evaluation, pulmonary artery and occlusion pressures, and the calculation of vascular resistance. The catheter is inserted into a central vein through an introducer port until it reaches the pulmonary artery. The introducer port, also known as the Cordis sheath, is a polyethylene sheath system with a hemostatic valve assembly and side port extension, which can act as a central vascular access point, theoretically eliminating the need for other CVAD in such patients.

## 5. Potential complications of central venous access

### Infectious sequelae

Prolonged dwell time leads to a statistically significant and exponential increase in infection rates, where the incidence of infection was found to be 4.8 per 1,000 catheter/days when dwell times were <10 days; such rates double when dwell times extend over a period of 20 days (72). Factors such as poor aseptic technique and catheter hub colonization also contribute to the risk of infection (24,33,37). Moreover, the experience level of the healthcare professional inserting the device may impact the potential risk of complications (73).

Central line associated blood stream infection (CLABSI) is a primary bloodstream infection occurring in a patient who has a central catheter in place at the time of (or within 48 h before) the development of the infection, and who does not have an infection at another site (74). The diagnosis of CLABSI does not necessarily require the central catheter to be the confirmed source of the bloodstream infection. Instead, it is an epidemiological surveillance definition used by the Centers for Disease Control and Prevention (CDC) to monitor the rates of these infections across healthcare institutions. CRBSI is a bloodstream infection where the catheter is confirmed as the source of the infection (75). The diagnosis of CRBSI typically requires more rigorous criteria compared to CLABSI. This usually includes a comparison of culture results from the catheter and a peripheral vein to demonstrate that the infection is more likely to have been caused by the catheter. It is pertinent to note that sending the catheter tip for culture offers poor positive predictive value (76)

In the USA, it is estimated that ~250,000 cases of CLABSI occur annually, contributing to the death of 12,000-28,000 patients and incurring billions in healthcare costs (75). Despite improvements in prevention, CRBSIs and CLABSIs remain a significant cause of healthcare-associated infections. The overall mortality rate associated with CRBSIs ranges from 12-25%, although it can be as high as 35% in intensive care units (77). Several risk factors for the development of CRBSIs and CLABSIs have been identified. These include the prolonged duration of catheterization, femoral or internal jugular placement (vs. subclavian), immunosuppression, inadequate staff education and training, poor adherence to infection control practices and improper catheter handling. The type of central access also accounts for infection risks; PICCs have been found to be associated with lower infection rates and longer time dwell times prior to colonization in critically ill patients when compared to other CICCs (58).

The most common pathogens associated with CRBSIs and CLABSIs include the following from the most common to the least common: Gram-positive organisms (coagulase-negative staphylococci, 34%; enterococci, 16%; and *Staphylococcus aureus*, 9.9%) and Gram-negative organisms (*Klebsiella*, 6%; *Enterobacter*, 4%; *Pseudomonas*, 3%; *Escherichia coli, 3%; Acinetobacter*, 2%) and yeast (*Candida* species, 12%) (72). The Infectious Disease Society of America (IDSA) recommends empiric treatment for CLABSI with vancomycin plus either piperacillin-tazobactam, cefepime, or a carbapenem. Definitive therapy is based on culture and susceptibility results. The duration of treatment varies based on the pathogen, although is typically between 7-14 days (78). In general, any catheters suspected of being the source of bacteremia or septicemia should be promptly removed. However, in some cases (e.g., infections with coagulase-negative staphylococci), catheter salvage may be attempted under certain conditions by using a combination of systemic antibiotics and antibiotic lock therapy. This is not recommended if there is hemodynamic instability or metastatic complications of infection. Consulting infectious disease specialists is recommended if cultures are positive for *Staphylococcus aureus* or *Candida* species, persistent bacteremia or shock after 72 h of appropriate antimicrobial therapy or presence of an intravascular prosthetic device (79).

Multiple strategies regarding mitigating the risk of infectious sequalae have been suggested. For instance, the American Society of Anesthesiologists Task Force on Central Venous Access endorses aseptic techniques and the use of maximal barrier precautions (80). Additionally, the use of chlorhexidine for skin preparation before catheter insertion has been shown to significantly reduce CRBSIs compared to povidone-iodine (81). Additionally, the use of chlorhexidine-containing dressings is currently considered an ‘essential practice’ (82). The use of antimicrobial coated catheters is highly recommended by multiple societies; this stems from multiple studies and a meta-analysis that demonstrated significant differences in the rate of CRBSIs per 1,000 catheter-days between antimicrobial-impregnated and standard CVADs (RR, 0.70; 95% CI, 0.53-0.91; P=0.008) (83).

### Catheter occlusion

Catheter occlusion is often encountered in CVADs and is most commonly due to thrombosis of the catheter (84). Other causes of occlusion include catheter malposition, migration, drug precipitation and mechanical occlusion. Lock solutions, such as heparin and citrate are left in catheters when not in use to decrease the risk of thrombosis. Citrate chelates calcium ions decreasing the activation of the coagulation cascade and platelet aggregation (85). Notably, the use of citrate is preferred in patients on hemodialysis as it has been shown to reduce the frequency of flow-related catheter exchanges. Citrate is also preferred in patients who develop heparin-induced thrombocytopenia. In cases of catheter thrombosis, alteplase or other thrombolytic agents, such as tenecteplase are often used to restore flow (84). Recently, taurolidine-based catheter lock solutions have shown promising results in decreasing both catheter-related thrombosis and infections, but are not currently commercially available (86). The diagnosis of catheter-related thrombosis is achieved with duplex ultrasonography. The treatment of catheter-related thrombosis is individualized; however, the mainstay of therapy is anticoagulation. Catheter removal is not indicated if the catheter is functioning appropriately and is still needed for patient care (87).

### Emerging trends and technologies

Recent advancements in VADs are revolutionizing clinical practice through improvements in VAD design and management. For instance, the application of high-strength, thromboresistant hydrogels has improved catheter flexibility and durability (88). Novel advancements in anti-infective coating, anti-infective VAD hubs, and novel needleless connectors, have been shown to decrease both infectious and mechanical complications (89). For instance, antimicrobial (minocycline, rifampin and chlorhexidine) Polycarbonate coating for intravenous connectors leads to the cessation of microbial colonization (90). Hydrophobic catheter material has been noted to be associated with a similar risk of VAD complications and failure when compared to standard polyurethane; however, hydrophilic catheter material has been shown to decrease thrombotic and infectious complications and improve patient comfort (91,92).

Furthermore, artificial intelligence (AI) has been employed to optimize catheter design; for example, AI-aided geometric modifications have been shown to significantly impede bacterial migration along catheter surfaces, reducing infection rates by up to 100-fold (93). AI-based models have also been used in accurately and reliably determining CVC placement in chest X-rays (94).

## 6. Conclusion

The present review provides clinicians with essential insight into selecting the most appropriate venous access device based on current evidence-based recommendations. The present review serves as a clinical decision-making aid through summarizing best practices and addressing potential knowledge gaps. Different access sites have both advantages and disadvantages based on the duration of therapy, flow rates and risks of complications. The selection of a vascular access device, as well as the insertion site should be thoughtfully approached based on indications, intended use, anticipated duration and specific patient factors. Adverse events, such as bloodstream infections and thrombosis, are key issues that can alter the trajectory of therapy and lead to poor patient outcomes. As venous access carries inherent risks, the prudent choice of device and meticulous procedural and post-procedural care is paramount in ensuring patient safety and successful therapy.

## Figures and Tables

**Table I:** Summary of various venous access devices.

Device type	Insertion site and tip location	Dwell time	Notes
Peripheral IV catheters	Peripheral veins	72-96 h	Ideal for short-term therapies; high failure rate and increased risk of phlebitis and mechanical complications with prolonged use.
Long peripheral catheters	Peripheral veins	Up to 4 weeks	Useful in patients with poor IV access; extended dwell time compared to PIVCs.
Midline catheters	Larger peripheral veins (e.g., basilic, brachial, or cephalic) with tip in axillary or subclavian vein	6-14 days	Lower incidence of phlebitis than PIVCs; contraindicated for vesicant medications.
Peripherally inserted central catheters	Peripheral veins (typically basilic or cephalic) with tip positioned at the cavoatrial junction	Several weeks to 6 months	Preferred for long-term IV therapies (e.g., antibiotics); reduces hospitalization costs, not recommended if therapy lasts <5 days.
Non-tunneled centrally inserted central catheters	Inserted in central veins (e.g., internal jugular, subclavian, or femoral) with tip in the central circulation	Days to 2 weeks	Employed in critical care settings for ease of insertion and multi-lumen access.
Tunneled centrally inserted central catheters	Inserted in central veins (e.g., internal jugular, subclavian, or femoral) with tip in the central circulation	Weeks to months	Tunneled catheters reduce infection risk and have more secure fixation. Can provide long term dialysis access.
Implantable ports	Surgically implanted on the chest wall accessing the subclavian vein with tip in central circulation	Months to years	Commonly used in oncology for chemotherapy; improve patient comfort and allow normal daily activities.

It should be noted that patient specific factors need to be taken into account. For instance, the anticipation of longer-term treatment favors earlier PICC placement, while those in resource limited resources would find insertion of long peripheral catheters more beneficial as no additional training is required unlike midlines or PICCs. IV, intravenous; PICCs, peripherally inserted central catheters.

## Data Availability

Not applicable.
